# Embryonic Ontogeny of 5-Hydroxyindoles and 5-Methoxyindoles Synthesis Pathways in the Goose Pineal Organ

**DOI:** 10.3390/ijms20163948

**Published:** 2019-08-14

**Authors:** Maria Hanuszewska, Magdalena Prusik, Bogdan Lewczuk

**Affiliations:** Department of Histology and Embryology, Faculty of Veterinary Medicine, University of Warmia and Mazury in Olsztyn, Oczapowskiego 13, 10-719 Olsztyn, Poland

**Keywords:** serotonin, melatonin, embryonic ontogeny, pineal organ, birds

## Abstract

The aim of this study was to characterize the embryonic ontogeny of 5-hydroxyindoles and 5-methoxyindoles synthesis pathways in the goose pineal organ. The study was performed on embryos aged 14–28 days, which have been incubated under a 12L:12D cycle. The pineal organs were collected for measurements of indole content by HPLC every 6 h on embryonic day (ED) 14, ED 16, ED 18 and ED 22 or every 2 h on ED 24, ED 26 and ED 28. The level of tryptophan showed no significant changes during development and no day-night variations. The content of 5-hydroxytryptophan increased between ED 14 and ED 26. It was significantly higher during scotophase than during photophase starting from ED 14. The serotonin content was low during the early stages of development (ED 14–ED 18) and prominently increased from ED 20. The serotonin levels also showed day-night differences; however, they were less conspicuous than those of 5-hydroxytryptophan. The changes in the level of 5-hydroxyindole acetic acid were similar to those of serotonin. 5-Hydroxytryptophol was measurable from ED 18. Levels of *N*-acetylserotonin, which were detectable for the first time on ED 16, prominently increased between ED 22 and ED 28 and showed significant day–night differences from ED 20. Melatonin was detectable from ED 18. Like *N*-acetylserotonin, its content increased rapidly between ED 22 and ED 28, and from ED 20 showed diurnal variations. 5-Methoxyindole acetic acid and 5-methoxytryptophol occurred at measurable levels from ED 18 and ED 26, respectively. The obtained results showed that embryonic development of indole metabolism in the goose pineal organ starts with the beginning of serotonin synthesis. The processes of serotonin acetylation and 5-hydroxyindoles methylation were turned on later. Diurnal rhythmicity develops very early in the embryonic pineal organ of the goose when the eggs are incubated under a 12 h light: 12 h dark schedule. Two processes are responsible for generation of the diurnal rhythms of 5-hydroxyindoles and 5-methoxyindoles: (i) hydroxylation of tryptophan and (ii) acetylation of serotonin.

## 1. Introduction

It is well documented that the synthesis of melatonin (MLT) starts in the avian pineal organ during the period of embryonic development and occurs in a diurnal rhythm when the eggs are incubated in an appropriate light-dark cycle [1,2,3,4,5,6,7]. This situation is different from that in the mammalian species investigated to date, in which the process of MLT secretion begins after birth [6,8,9]. In vitro studies demonstrated that MLT secretion from the pineal organs of chicken embryos is controlled by environmental light and the intrapineal circadian oscillator [10,11,12,13,14,15,16,17]. Furthermore, embryonic chicken pinealocytes respond with an increase in MLT secretion to some neuropeptides, including vasoactive intestinal peptide [13,18,19]. However, it should be stressed that our knowledge of the embryonic ontogeny of MLT synthesis is still largely incomplete because published research has focused only on some steps of this biochemical pathway. The majority of in vivo studies concerned the expression and activity of arylalkylamine *N*-acetyltransferase (AA-NAT) and *N*-acetylserotonin *O*-methyltransferase (ASMT) [3,20,21,22,23,24,25,26,27,28]. These enzymes play a crucial role in the regulation of MLT synthesis as an MLT rhythm-generating factor (AA-NAT) and an MLT-synthesis limiting factor (ASMT). However, the data on the levels of mRNAs encoding the enzymes and their activity measured in the in vitro assay do not answer the question about the actual level of MLT synthesis in embryos. This answer is provided by only a few studies, in which MLT levels were measured by radioimmunoassay [1,2,3,4,5,7]. A relatively large number of studies on MLT secretion by the embryonic chicken pineal organ were performed in vitro and they were focused mainly on the regulatory mechanisms [10,11,12,13,14,15,16,17]. In some cases, the incubation of the pineal organs in vitro was used to estimate the synthesis of MLT at early stages of development [10,11,14,15,29]. It should also be noted that the current state of knowledge about the pineal organ ontogeny in birds is the result of studies conducted on chicken embryos and, to a smaller degree, Japanese quail embryos [26,27,28,30]. Research on other species has been scarce [31]. The pineal glands of birds differ much in histological structure and ultrastructure, the content and daily profiles of indolic compounds, and the role of particular mechanisms in the regulation of melatonin secretion [32,33,34,35,36,37,38,39,40]. Significant interspecies differences indicate the necessity to conduct comparative research.

The MLT synthesis pathway is only one part of 5-hydroxy- and 5-methoxyindole metabolism in the pineal organ. This metabolism starts with hydroxylation of tryptophan (TRP) to 5-hydroxytryptophan (5-HTRP) catalyzed by tryptophan hydroxylase. Next, 5-HTRP is decarboxylated by aromatic amino acid decarboxylase to serotonin (5-HT). In the MLT synthesis pathway, 5-HT is acetylated by AA-NAT to *N*-acetylserotonin (NAS), which is methylated to MLT in a reaction catalyzed by ASMT. Another important metabolic pathway of pineal indole metabolism commences with oxidative deamination of 5-HT to 5-hydroxyindole acetaldehyde, an unstable compound, which is reduced to 5-hydroxytryptophol (5-HTOL) or dehydrogenated to 5-hydroxyindole acetic acid (5-HIAA). These 5-hydroxyindoles are methylated by ASMT to 5-methoxytryptophol (5-MTOL) and 5-methoxyindole acetic acid (5-MIAA), respectively. The next pathway of 5-HT transformation consists of methylation by ASMT to 5-methoxytryptamine (5-MTAM).

Studies covering all the compounds and processes mentioned above are infrequent; however, their results have clearly demonstrated that avian pineal organs differ concerning the content of indoles and the quantitative proportion between them [37,39,40]. For example, the content of serotonin 5-HT is high in the goose and the duck, and low in the chicken [37,39,40]. Simultaneously, the ratio of 5-HIAA to 5-HT in the pineal organ is approximately 10 in chickens and approximately 0.1 in geese. Differences in proportions between 5-hydroxyindoles are responsible for variability in the mechanisms involved in generation of the diurnal rhythm in the content of 5-MIAA and 5-MTOL between the chicken and the duck [37,39]. 

The aim of our study was to characterize the embryonic ontogeny of 5-hydroxyindoles and 5-methoxyindoles synthesis pathways in the goose pineal organ by analyzing changes in the content of these compounds. The domestic goose was chosen as a study object because of (i) the characteristic metabolic profile of pineal indoles with a high level of 5-HT in 14-week-old birds, (ii) the histological organization of the pineal organ in newly hatched geese, which is very similar to those in 14-week-old birds, and (iii) the long period of embryonic development (29 days vs. 21 days in the chicken). The study covered the period between the 14th (embryonic day 14, ED 14) and 28th day (ED 28) of egg incubation. The starting point was determined by the developmental stage at which the pineal organ can be precisely separated from the neighboring tissues. To characterize the ontogeny of the diurnal rhythmicity in pineal indole metabolism, the hatching eggs were incubated under 12 L:12 D cycle (similar to the natural cycle during breeding season in geese) and the pineal glands were taken every 6 h on ED 14, ED 16, ED 18, ED 20 and ED 22, and every 2 h on ED 24, ED 26 and ED 28.

## 2. Results

### 2.1. Content of Tryptophan, 5-Hydroxy- and 5-Methoxyindoles in the Pineal Organ during Embryonic Development

**Figure 1:**
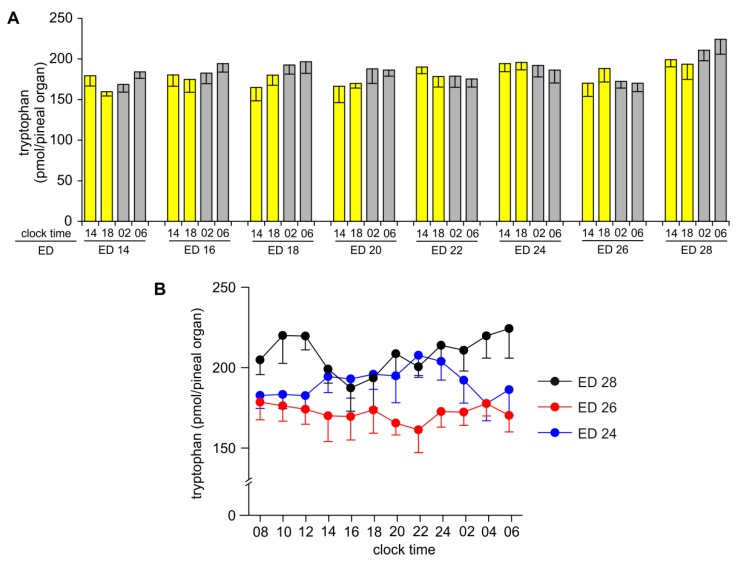
Content (means and SEM, *n* = 5) of tryptophan in the pineal organs of geese embryos between embryonic day (ED) 14 and ED 28 analyzed at 4 time points per day (**A**) and analyzed at 12 time points per day on ED 24, ED 26, and ED 28 (**B**).

The pineal content of TRP did not change significantly with the age of the embryos, nor did it show any significant time-dependent variations (Figure 1A,B).

**Figure 2:**
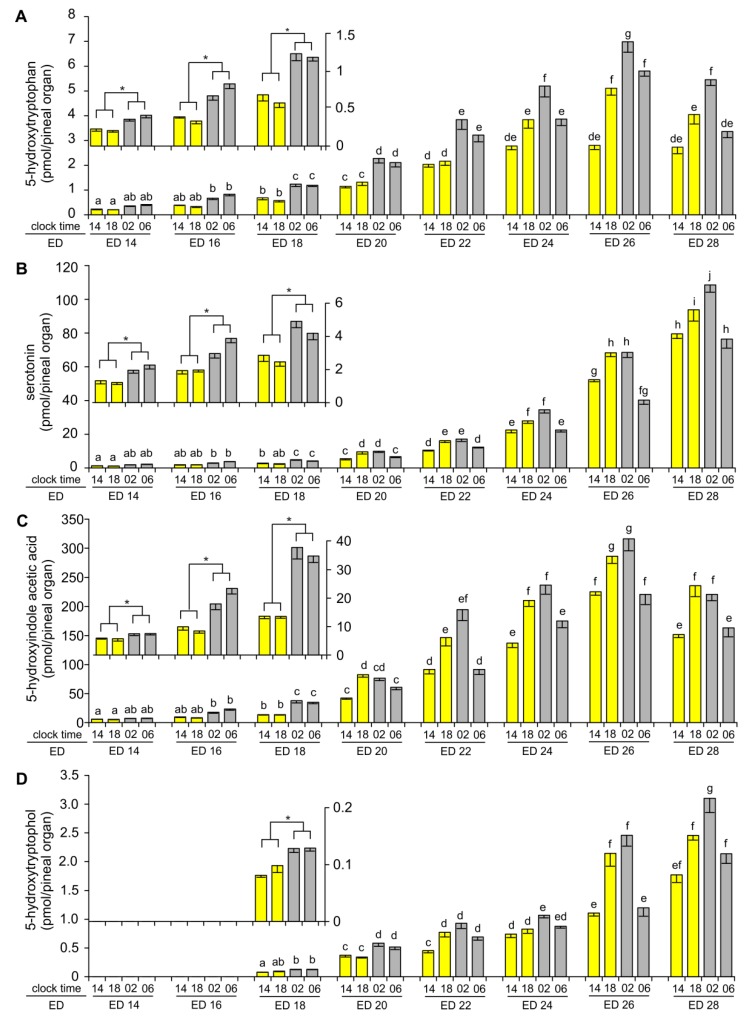
Content (means and SEM, *n* = 5) of 5-hydroxytryptophan (**A**), serotonin (**B**), 5-hydroxyindole acetic acid (**C**) and 5-hydroxytryptophol (**D**) in the pineal organs of geese embryos. Values labeled with different letters are significantly different in Duncan’s test performed as a post hoc procedure after two-way ANOVA. Inserts: Enlarged parts of charts showing data from ED 14, ED 16 and ED 18 embryos. Significant differences between sampling time points as showed by one-way ANOVA and Duncan’s test were signed with asterisk (*).

The content of 5-HTRP, measured in the middle of photophase at 14:00, increased stepwise between ED 14 and ED 24, and then remained unchanged (Figure 2A). The levels of 5-HTRP during scotophase, at 02:00 and at 06:00, increased between ED 14 and ED 26 and then decreased at ED 28. The content of 5-HTRP was significantly higher during scotophase, at 02:00 and 06:00, than during photophase at 14:00, at all investigated stages of embryonic development. Changes in the content of 5-HTRP measured at 18:00 were more complex. The levels of this compound were similar to those measured at 14:00 between ED 14 and ED 22. Later, on ED 24, ED 26 and ED 28, they were significantly higher at 18:00 than at 14:00.

The content of 5-HT in the embryonic pineal organs on ED 14, ED 16 and ED 18 was relatively low when compared to later periods of development (Figure 2B). It was significantly higher at 02:00 and 06:00 than at 14:00 and 18:00. Both day-time and night-time levels of 5-HT slightly increased with the age of the embryos during this period. The increase in the pineal content of 5-HT was much faster starting from ED 20. The level of 5-HT measured at 14:00 was 14-fold higher on ED 28 than on ED 20. Like in younger embryos, significant time-dependent changes in the content of 5-HT were noted, however the course of these changes was different. The level of 5-HT was significantly higher at 18:00 and 02:00 than at 14:00 and 06:00.

As in the case of 5-HT, the content of 5-HIAA at all studied time-points increased slowly between ED 14 and ED 18 (Figure 2C). During this period, it was significantly higher at 02:00 and 06:00 than at 14:00 and 18:00. A prominent increase in the level of 5-HIAA occurred between ED 20 and ED 26. On ED 28, the content of 5-HIAA was lower than at ED 26 at all investigated time-points. On ED 24–ED 28, the level of 5-HIAA was higher at 18:00 and 02:00 than at 14:00 and 06:00.

5-HTOL was undetectable in the pineal organs taken on ED 14 and ED 16 (Figure 2D). The content of 5-HTOL was only slightly above the quantification limit on ED 18; however, it increased prominently during the following two days, being 4–5 times higher at ED 20 than ED 18. Next, the level of this compound increased up to the end of incubation, with a remarkable surge on ED 26 and ED 28. The level of 5-HTOL was significantly higher at 02:00 and 06:00 than at 14:00 and 18:00 on ED 18–ED 20. From ED 22, the content of 5-HTOL was significantly higher at 18:00 and 02:00 than at 14:00 and 06:00.

**Figure 3:**
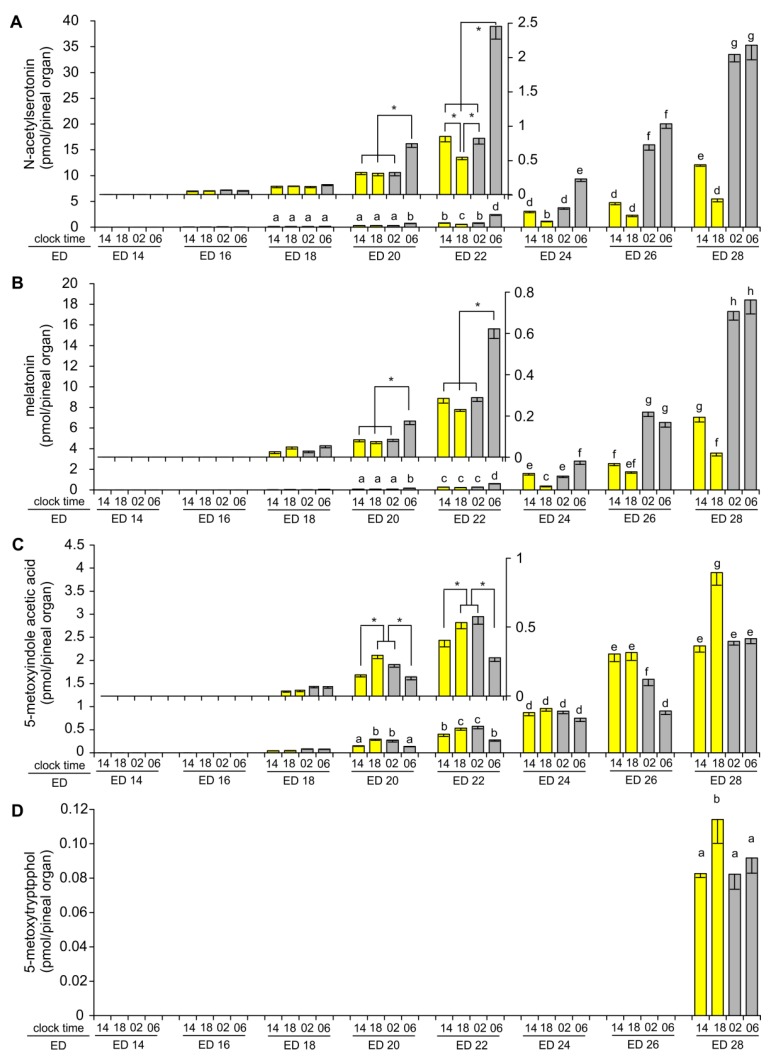
Content (means and SEM, *n* = 5) of *N*-acetylserotonin (**A**), melatonin (**B**), 5-methoxyindole acetic acid (**C**) and methoxytryptophol (**D**) in the pineal organ of geese embryos. Values labeled with different letters are significantly different in Duncan’s test performed as a post hoc procedure after two-way ANOVA. Inserts: Enlarged parts of charts showing data from ED 14, ED 16 and ED 18 embryos. Significant differences between sampling time points as showed by one-way ANOVA and Duncan’s test were signed with asterisk (*).

In the course of embryonic development NAS was detectable for the first time in the goose pineal organ on ED 16 (Figure 3A). The level was relatively low between ED 18 and ED 22, despite a continuous increase with the age of the embryos. Significant differences in the level of NAS between sampling time-points were noted for the first time on ED 20, when the content of NAS was higher at 06:00 than at 14:00, 18:00 and 02:00. On ED 22, the level of NAS was also the highest at 06:00 but significantly lower at 18:00 than at 14:00 and 02:00. Higher levels of NAS and more pronounced diurnal variations were noted from ED 24. The course of daily changes in the level of NAS on ED 24 was similar to that on ED 22. It was different on ED 26 and ED 28, when the level of NAS was significantly higher at 02:00 and 06:00 than at 14:00 and 18:00, as well as at 14:00 than at 18:00.

MLT was undetectable on ED 14 and ED 16, hardly detectable on ED 18 and only slightly extended the limit of quantification on ED 20 (Figure 3B). The content of MLT increased quickly between ED 22 and ED 28. The level of MLT on ED 20 and ED 22 was significantly higher at 06:00 than at 14:00, 18:00 and 02:00. On ED 24, the MLT level was significantly higher at 06:00 than at 14:00, 18:00 and 02:00 and at 14:00 and 02:00 than at 18:00. On ED 26 and ED 28, the MLT level was significantly higher at 02:00 and 06:00 than at 14:00 and 18:00, and at 14:00 than at 18:00.

5-MIAA was detected for the first time on ED 18, and its level increased during subsequent days of embryonic development (Figure 3C). The level of 5-MIAA was significantly higher at 18:00 and 02:00 than at 14:00 and 06:00 on ED 20 and ED 22. No significant variations between analyzed time points were noted on ED 24. On ED 26, the level of 5-MIAA was higher at 14:00 and 18:00 than at 02:00 and 06:00, as well as at 02:00 than at 06:00. On ED 28, the level of 5-MIAA was higher at 18:00 than at 14:00, 02:00, and 06:00.

5-MTOL was detected in the goose pineal organs exclusively on ED 28 (Figure 3D). 5-MTAM was undetectable.

### 2.2. Diurnal Profiles of 5-Hydroxy- and 5-Methoxyindoles in the Embryonic Pineal Organs on ED 24, ED 26 and ED 28

**Figure 4:**
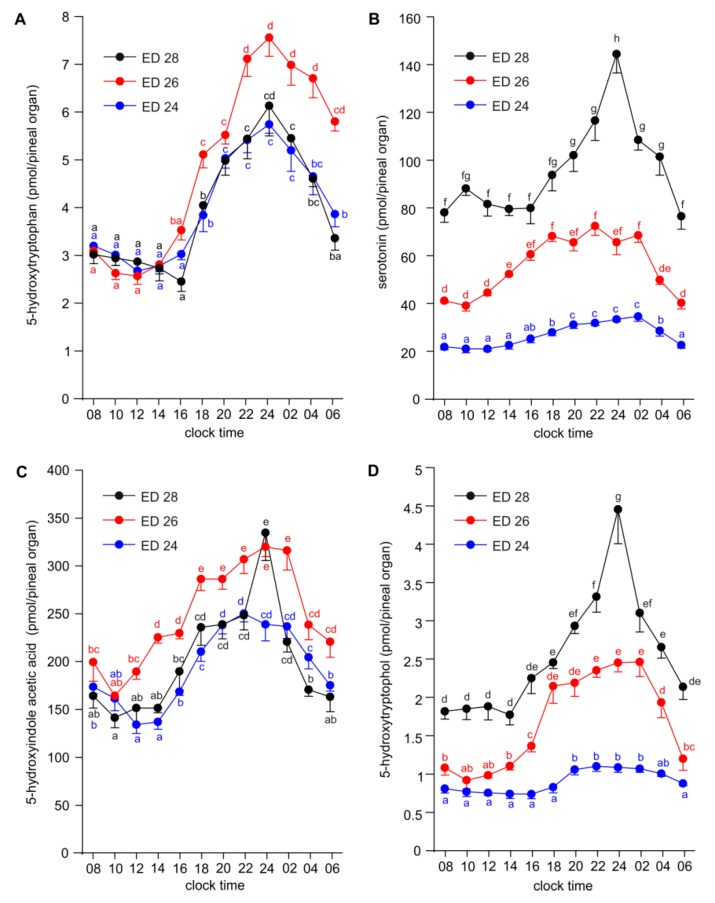
Diurnal changes in the content of 5-hydroxytryptophan (**A**), serotonin (**B**), 5-hydroxyindole acetic acid (**C**) and 5-hydroxytryptophol (**D**) in the pineal organs of geese embryos on ED 24, ED 26 and ED 28 (means and SEM, *n* = 5). Values labeled with different letters are significantly different.

The pineal content of 5-HTRP was at a stable level between 08:00 and 16:00, increased stepwise up 24:00, and later decreased at a similar rate as it had increased on ED 24–ED 28 (Figure 4A). The night-time increase in 5-HTRP level was higher in the pineal organs from the embryos taken on ED 26 than on ED 24 and ED 28.

The level of 5-HT differed significantly at all sampling time points between the pineal organs taken on ED 24, ED 26 and ED 28, being higher in older embryos (Figure 4B). On ED 24, the content of 5-HT was rather stable between 08:00 and 14:00, then slowly increased to achieve the maximum at 02:00 and later quickly decreased. The pineal content of 5-HT in ED 26 embryos increased slowly between 10:00 and 18:00, then showed a plateau until 02:00 and then declined between 02:00 and 06:00. On ED 28, an even, low content of 5-HT was found between 08:00 and 16:00, then 5-HT levels increased to reach the maximum at 24:00 and later decreased until 06:00.

The diurnal rhythm of 5-HIAA content was found in the pineal organs of ED 24, ED 26 and ED 28 embryos (Figure 4C). The courses of daily changes in 5-HIAA levels were similar to those observed in a case of 5-HT. The lowest levels of 5-HIAA were noted in the first half of photophase and the highest in the first half of scotophase. A prominent drop in 5-HIAA levels occurred in the second half of scotophase. This drop took place earlier and was more pronounced in embryos taken at ED 28 than in those taken at ED 26 and ED 24. The difference in the level of 5-HIAA between ED 24, ED 26 and ED 28 embryos was much lower than in the case of 5-HT.

The level of 5-HTOL showed significant diurnal variations, which were generally similar to those of 5-HIAA, in all studied groups of embryos (Figure 4D). The amplitudes of the 5-HTOL daily rhythms were slightly higher than those of the 5-HIAA rhythms in ED 24 and ED 26 embryos. On ED 28, both rhythms were parallel. During the whole sampling period, the content of 5-HTOL differed prominently between the pineal organs of ED 24, ED 26 and ED 28 embryos.

**Figure 5:**
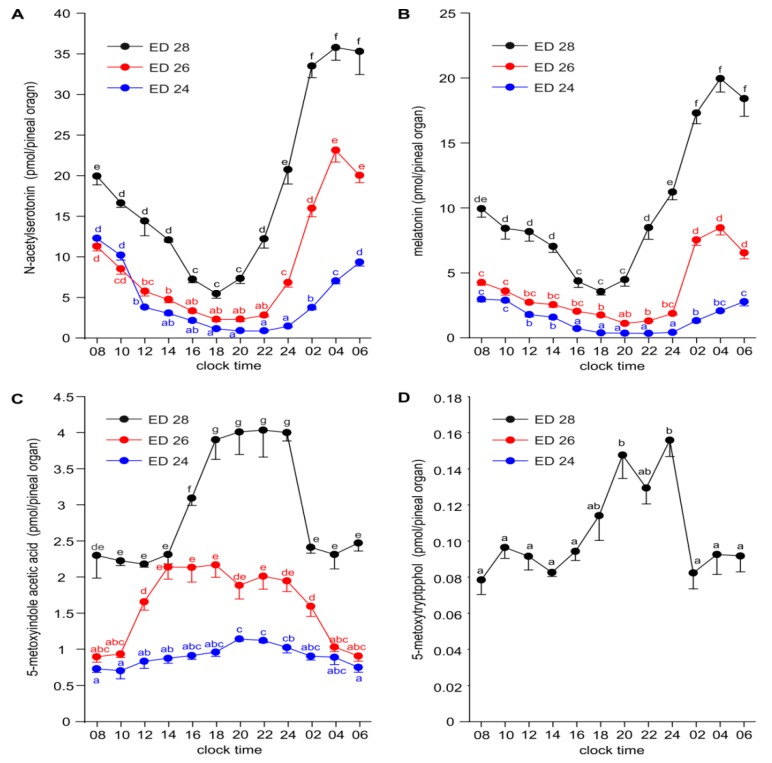
Diurnal changes in the content of *N*-acetylserotonin (**A**), melatonin (**B**), 5-methoxyindole acetic acid (**C**) and methoxytryptophol (**D**) in the pineal organs of geese embryos on ED 24, ED26 and ED 28 (means and SEM, *n* = 5). Values labeled with different letters are significantly different.

The diurnal rhythms of NAS content were characterized by the highest differences between the minimum and maximum levels among the investigated indoles (Figure 5A). The level of NAS significantly increased with the age of the embryos. The course of the diurnal rhythms of NAS differed between ED 24, ED 26 and ED 28 embryos by the times of peak and nadir occurrence. NAS levels were lowest at 20:00 and 22:00, and highest between 06:00 and 10:00 in the pineal organs of ED 24 embryos. In ED 26 embryos, the minimum and maximum levels occurred earlier than in younger embryos, at 18:00–20:00 and at 04:00, respectively. The diurnal rhythm of NAS in ED 28 embryos was characterized by the nadir at 18:00 and the peak between 02:00 and 04:00. The amplitude of the NAS rhythm decreased with the age of the embryos (12 vs. 10 vs. 6.5).

The diurnal rhythms of MLT content were similar to those of NAS with the exception of lower amplitudes. The levels of MLT differed prominently between ED 24, ED 26 and ED 28 embryos (Figure 5B).

The content of 5-MIAA showed diurnal rhythms in ED 24, ED 26 and ED 28 embryos; however, the course of these rhythms varied according to the age of the embryos (Figure 5C). The level of this compound slowly increased during photophase and slowly decreased during scotophase in ED 24 embryos. In ED 26 embryos, it increased rapidly between 10:00 and 14:00, then remained stable up to 24:00 before decreasing. In ED 28 embryos, the level of 5-MIAA was low between 08:00 and 14:00, rapidly increased between 14:00 and 18:00, remained at a high stable level up to 24:00 and then quickly decreased between 24:00 and 02:00 to the level occurring in the first half of photophase. The rhythms of 5-MIAA were not parallel to the rhythms of 5-HIAA, a direct precursor of 5-HIAA, especially during the photophase in ED 24 and ED 26 embryos.

5-MTOL was detectable exclusively in ED 28 embryos (Figure 5D). Its level was the highest in the first half of scotophase, between 20:00 and 24:00.

### 2.3. Changes in Proportions Between Indoles during Embryonic Development

**Figure 6:**
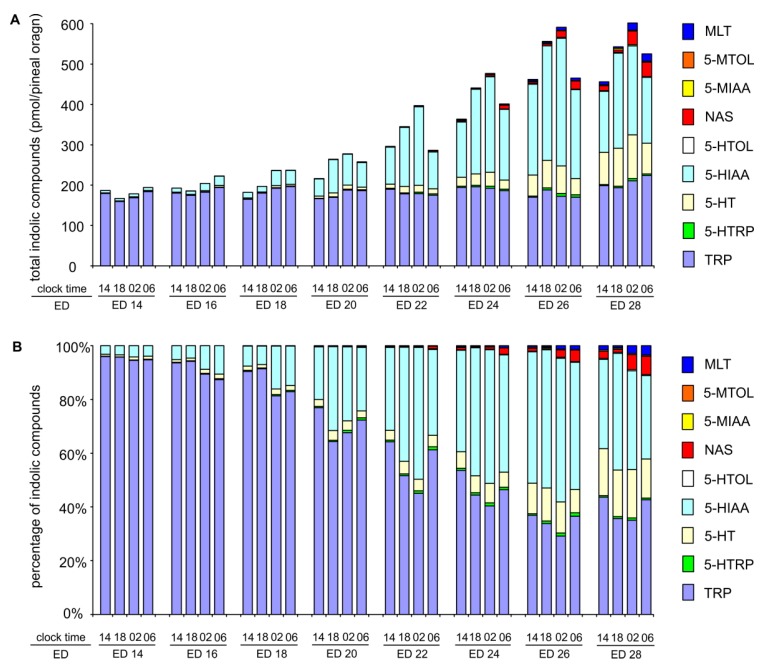
Changes in the stoichiometric relationships between indoles in the geese pineal organ during embryonic development expressed as absolute values (**A**) and percentages (**B**).

As described in Section 2.1., the contents of 5-hydroxy- and 5-methoxyindoles in the goose pineal organ increased between ED 14 and ED 26, while the level of TRP remained constant (Figure 6A). The changes in the content of indoles are accompanied by changes in the proportions between them, which was demonstrated in Figure 6B.

## 3. Discussion

The present study characterizes, for the first time, the embryonic ontogeny of 5-hydroxyindoles and 5-methoxyindoles biosynthesis pathways in the avian pineal organ based on measurements of the content of these compounds.

The obtained data enable us to distinguish three phases of development of indole metabolism in the embryonic goose pineal gland. During the first phase, the pineal organ produces and metabolizes 5-HT, but it lacks the (measurable) ability to perform 5-HT acetylation and methylation of 5-hydroxyindoles. This phase is represented in our study by the pineal organs of ED 14 embryos. Unfortunately, the studied material did not cover the time point when the process of 5-HT synthesis starts; however, very low levels of TRP derivatives, as well as, a low share of 5-HTRP (<0.25%), 5-HT (<1%) and 5-HIAA (<3.75%) and a high share of TRP (95–96%) among the studied indoles suggest that ED 14 is very close to this moment. We did not study the pineal organs of younger embryos because of the problem with their precise dissection from the surrounding tissues. Two ideas, which are not mutually exclusive, should be considered concerning the significance of 5-HT synthesis at this phase of development. The first considers this phase as a phylogenetically determined step in the ontogenesis of the MLT-synthesis pathway. According to the second idea, 5-HT plays a role as a paracrine or/and endocrine agent in embryos. Attention should be paid to relatively high levels of 5-HIAA, which demonstrates that large amounts of 5-HT undergo oxidative deamination. During the second phase of indole metabolism development, the process of 5-HT acetylation is turned on. The pineal organ produces NAS, but it is not able to produce MLT at all or at a measurable level. This phase occurred around ED 16 in the goose pineal organ. The last, third phase in the development of indole metabolism starts with the ability to methylate 5-hydroxyindoles. We detected 5-methoxyindoles (5-MIAA and MLT) for the first time in the pineal organs of ED 18 embryos, but their levels were very low. The contents of both 5-MIAA and MLT increased prominently with the age of embryos.

As mentioned in the Introduction, there are no published data on indoles other than MLT in the embryonic pineal organ of birds. The starting point of MLT synthesis in the chicken pineal organ has been proposed to occur about ED 10–ED 11 [29]. In the experiment, in which the chicken pineal organs were incubated in vitro for 24 h, trace amounts of MLT that accumulated in the medium (approximately 5 pg/24 h) were found in ED 10 (but MLT was undetectable in the pineal organ at this time). The release of MLT was higher in ED 11 and slowly increased during the following days. A prominent increase in MLT release was noted in this experiment as late as between ED 17 and ED 19 [29]. These results agree with the detection of trace ASMT activity in the pineal organs of 10- or 12-day-old embryos [20,25] and trace NAT activity in 11- or 14-day-old embryos [22,23]. Regarding ASMT, mRNA encoding this enzyme was detected in the chicken pineal organ for the first time during development at ED 8 by RT-PCR and at ED 12 by northern blotting [25]. For comparison, ASMT protein was detectable at ED 17 [24]. The release of MLT in superfusion culture from the chicken pineals was demonstrated at ED 13; however, the release was approximately 10-fold lower than that from the organs at ED 18 [10]. It should be noted that the majority of data about MLT synthesis in the embryonic pineal organ were obtained from in vitro experiments. Therefore, little is known about the in vivo levels of this pineal hormone. According to our unpublished data, isolation of the pineal organ from the body and an artificial environment can influence indole metabolism in embryonic tissues. Summing up the current stage of knowledge, MLT synthesis occurs in the chicken pineal organ in the second half of embryonic life. Its level is initially very low and greatly increases around ED 18–ED 19.

The differences in the length of incubation period and development [41] should be taken into consideration during comparison of the embryonic ontogeny of MLT synthesis in the goose and the chicken. The process of MLT synthesis starts in both species at similar phases of embryonic development. However, in the goose pineal organ large amounts of MLT are present approximately six days before hatching, while in the chicken pineal organ the content of MLT rises later, around ED 18–ED 19. As mentioned above, the data on in vivo levels of MLT in embryonic avian pineal organs are still incomplete and further comparative studies are needed for better understanding of ontogenesis of the pineal hormone synthesis in birds.

A well-documented feature of the avian pineal organ is the presence of rhythmical changes in indole metabolism. Our results show that diurnal rhythmicity develops very early in the embryonic pineal organ of the goose when the eggs are incubated under a 12 h light: 12 h dark schedule. We found that the pineal content of 5-HTRP, 5-HT and 5-HIAA show diurnal variation in ED 14 embryos, being higher during scotophase than during photophase. These variations are obviously driven by changes in the activity of tryptophan hydroxylase, an enzyme that can be controlled at the transcriptional level by clock genes and at the posttranscriptional level by several, various factors [36,42,43,44,45]. The diurnal rhythms of mRNA levels and the activity of tryptophan hydroxylase during the posthatching period have been described in the chicken pineal gland [36,42,43,44,45]. The daily variations in the level of 5-HTRP were reported in the pineal organs of 16-day-old chicken, 14-week-old ducks and 14-week-old geese [37,39,40]. In the goose, the rhythmical changes in 5-HTRP were endogenously generated in continuous darkness [40]. The present study shows that the course of daily variations in 5-HTRP content slightly changes during embryonic development of the goose pineal organ. Between ED 14 and ED 22, the level of 5-HTRP is higher at 02:00 and 06:00 than at 14:00 and 18:00; however, between ED 24 and ED 28, the level of 5-HTRP is higher at 18:00, 02:00 and 06:00 than at 14:00. The detailed analysis of the diurnal profiles of 5-HTRP in the pineal organs of ED 24, ED 26 and ED 28 embryos shows that the level of this compound is low up to 16:00, then increases up to 24:00 and later decreases to the daytime value.

According to our data, the daily changes in 5-HT content are very close to those of 5-HTRP in the pineal organs of ED 14–ED 18 embryos, when NAS synthesis is lacking or very low. Some discrepancies between these two compounds occur between ED 20 and ED 28, which are probably caused by large changes in NAS synthesis taking place at these developmental stages. It should be noted that the 5-HT content is several times higher than the level of 5-HTRP, and changes in the mechanism of 5-HT storage can also be responsible for discrepancies between daily profiles of 5-HTRP and 5-HT. The diurnal profiles 5-HIAA and 5-HTOL level are parallel to the changes in 5-HT content.

It is generally accepted that the diurnal rhythm of MLT secretion is driven by changes in the intensity of 5-HT acetylation into NAS, catalyzed by AA-NAT [3,22,23,26,27,28,34]. In our study, significant diurnal variations in the pineal content of NAS were noted for the first time in ED 20 embryos, in which the level of this compound was 3-fold higher at 06:00 than at 14:00, 18:00 and 02:00. During the following days, the peak diurnal rhythm of NAS level shifted backwards, i.e., from the transition period between scotophase and photophase in ED 22 and ED 24 embryos to the second half of scotophase in ED 28 embryos. The amplitude of NAS diurnal variations increased between ED 20 and ED 24 and then slightly decreased. The course of diurnal changes in the pineal MLT followed the NAS rhythm; however, the peak and the amplitude of these changes were lower than those of NAS. The lower peak of MLT compared to that of NAS points to the limiting effect of ASMT. Recently, several studies demonstrated that the activity of ASMT is too low to transform the entire amount of NAS formed during scotophase into MLT, both in birds and in mammals [37,39,46,47].

The embryonic development of MLT secretion rhythmicity was intensively studied in the chicken pineal organ, mainly using various in vitro systems [10,11,12,13,14,15,16,17]. The diurnal rhythm of MLT release with an increase during the dark phase was described in the pineal cell cultures from 13- and 14-day-old chick embryos performed in a 12 L:12 D cycle [10]. The rhythm reversed after the light-dark cycle had been reversed [10]. However, a rhythm of MLT release was not detected under constant dark conditions in a cell culture from these embryos [10]. These data clearly show the chicken pineal organ is photosensitive around ED 13, but lacks a working circadian oscillator [10,13,15]. Further research demonstrated that the circadian clock in the chicken pineal organ develops at 16–18 embryonic days [5,13,14,48]. In vivo, an MLT rhythm was found in the pineal organs of 19-day-old chick embryos maintained under 12 L:12 D and 16 L:8 D, but not under 8 L:16 D [2]. Likewise, light-dependent rhythmic transcription of AA-NAT mRNA was detected in the pineals of 16- and 19-day-old chick embryos [3,17]. Diurnal rhythm of AA-NAT activity was revealed at ED 17, 18 and ED 19 [21,23].

The diurnal changes in the AA-NAT activity have more extensive effects on the pineal indole metabolism than generation of the MLT secretion rhythm itself [37,39,46,47,48,49,50]. The nighttime increase in AA-NAT activity usually induces declines in the levels of 5-HTRP, 5-HT, 5-HIAA and 5-HTOL; however, these effects differ between species, being dependent on the quantitative proportions between indoles and the magnitude of the nighttime increase in NAS synthesis [37,39,40]. Our data show opposite diurnal variations in the level of NAS and the contents of 5-HTRP, 5-HT and 5-HIAA in the pineal organs of ED 24, ED 26 and ED 28 embryos. Moreover, they demonstrated that the developmental changes in the patterns of the NAS rhythm are followed by modification of the daily profiles of 5-HTRP, 5-HT and 5-HIAA.

The daily rhythm of AA-NAT may also affect the synthesis of 5-MIAA and 5-MTOL due to the competition between NAS, 5-HIAA and 5-HTOL for the catalytic activity of ASMT. Previous studies showed that this mechanism is solely responsible for generation of the diurnal rhythms of 5-MIAA and 5-MTOL in the chicken pineal organ [39], while it only slightly influences the synthesis of both 5-methoxyindoles in the duck pineal organ [37]. The results of present study show that the level of 5-MIAA increases faster than the level of its precursor, 5-HIAA, during the first half of photophase, when the content of 5-hydroxyindoles is probably below the catalytic capacity of ASMT. The proportion of 5-HIAA methylation declines with an increase in the content of 5-HIAA during the second half of photophase and an increase in NAS during scotophase.

The increase in biosynthesis of 5-hydroxyindoles and 5-methoxyindoles during the embryonic development of the pineal organ resulted in prominent changes in the quantitative proportions between indolic compounds. The share of TRP decreased from approximately 95% in ED 14 to less than 50% in ED 28, and this value is similar to that found in 14-week-old geese. However, it should be underlined that the composition of pineal indoles at the end of embryonic life differs markedly from that in 14-week-old geese, especially concerning 5-HT and 5-HIAA [40]. The share of 5-HT among the investigated pineal indoles increased prominently at the end the of embryonic development and reached 13–16% at ED 28. Nevertheless, this value is more than 3-fold lower than the percentage of 5-HT in the pineal organs of 14-week-old geese, where 5-HT represents 40–60% of indoles, depending on the phase of the daily cycle. Conversely, the percentage content of 5-HIAA decreased slightly between ED 24 and ED 28 to reach 33-43%. This value extends by several fold the shares of 5-HT metabolites in the total amount of pineal indoles in 14-week-old geese, in which the percentage content of 5-HIAA does not extend beyond 6%. Our results suggest that the mechanism of 5-HT protection against oxidative deamination and 5-HT storage starts to develop during embryonic life, but this process also occurs post-hatching.

Previous studies showed that metabolic profiles of the pineal indoles differ significantly between avian species [37,39,40]. The goose and duck pineal organs contain high levels of 5-HT and low levels of 5-HIAA and 5-HTOL [37,40]. Conversely, the chicken pineal organ is characterized by a low level of 5-HT and high levels of 5-HIAA and 5-HTOL [39]. When the same group of pineal indoles as in the present study was measured using our methodology in 16-day-old chickens, it was found that 5-HT represents only from 1 to 2% of indoles and 5-HIAA—from 18 to 38%. Comparison of these data with the results of the present study (13–16% of 5-HT in ED 28 embryos) suggests that the type of pineal indole metabolic profile, with respect to 5-HT levels is determined during embryonic development.

## 4. Materials and Methods

### 4.1. Egg Incubation

Hatching eggs of the domestic goose (*Anser anser f. domestica*) were incubated in Ova-Easy 580 Advance Series II cabinet incubator (Brinsea, Titusville, FL, USA) at a temperature of 37.0–37.8 °C and relative humidity of 55–75% (see Table 1 for details) in a 12 h light: 12 h dark cycle. During the day (07:00–19:00), the eggs were illuminated with white light (5000 K) emitted by LED lamps mounted to the external surface of the translucent door of the incubator. The light intensity measured on the surface of the eggs varied from 15 to 20 lx. At night, the incubation was conducted in complete darkness. Eggs were turned automatically around the long axis every hour (45°) and manually around the short axis every day (180°). Starting from ED 16, the incubators were left open for 15 min, and thereafter, the eggs were sprinkled with water to mimic the natural behavior of a goose sitting on the egg. This procedure was performed twice a day, at 10:15 and 16:15.

### 4.2. Tissue Collection

During the scotophase, eggs were taken from the incubator in complete darkness and transferred to the neighboring room, where decapitation was immediately performed under a dim red light with an intensity below 3 lx. The heads were prepared for opening the skull and placed under a stereomicroscope equipped with custom made red illumination with an intensity of 30 lx (Stemi DV4, Carl Zeiss, Oberkochen, Germany). Then, the microscope illumination was turned on, the pineal organs were excised and frozen at −80 °C within less than 45 s of exposition to the illumination. During photophase, the procedure was similar to that described above, but it was performed under white light with an intensity approximately 30 lx. Five pineal organs per time-point from each age-group were collected. All experimental procedures on embryos were performed in accordance with Polish law (project No 2018/31/N/NZ4/01248, 15 January 2015).

### 4.3. Analytical Procedure

#### 4.3.1. Chemicals

Sodium acetate, disodium EDTA (Ethylene Diamine Tetraacetic Acid) and acetic acid (all of the highest chemical purity) were purchased from J.T. Baker Chemicals (Phillipsburg, NJ, USA). Methanol of gradient-grade HPLC purity and perchloric acid were obtained from Merck Millipore (Billerica, MA, USA). TRP, 5-HTRP, 5-HT, NAS, MLT, 5-HIAA, 5-MIAA, 5-MTOL, and 5 MTAM were bought from Sigma-Aldrich (St. Louis, MO, USA). 5-HTOL was obtained from Santa Cruz Biotechnology (Dallas, TX, USA). Ultrapure water (18.2 MΩ, TOC ≤ 5 ppb) was freshly prepared using a Milli-Q Integral purification system (Merck Millipore, Billerica, MA, USA) and used in all analytical procedures.

#### 4.3.2. Sample Preparation

The pineal glands were sonicated in 80 μL of ice-cold 0.1 M perchloric acid using a Vibra-Cell VC 70 ultrasonic processor equipped with a 2-mm probe (Sonics & Materials Inc., Newtown, CT, USA). Next, the homogenates were incubated for 15 min in an ice-bath and centrifuged at 60,000 *g* (4 °C) for 15 min (Allegra 64R, Coulter Beckman, Indianapolis, IN, USA). The supernatants were transferred into appropriate plastic autosampler vials (La-Pha-Pack Werner Reifferscheidt GmbH, Langerwehe, Germany).

#### 4.3.3. HPLC Assay

Indole content was measured using a highly sensitive HPLC method based on gradient-grade separation and fluorescence detection with programmed sensitivity changes [37]. The chromatographic system was comprised of an LPG 3400A pump with a built-in degasser, WPS 3000SL autosampler, TCC 3100 column thermostat, and FLD 3400RS fluorescence detector (Dionex, Sunnyvale, CA, USA). The separation of indoles was performed at 30 °C using a Hypersil GOLD aQ column 150 × 4.6 mm, 3-μm particle size (Thermo Scientific, Waltham, MA, USA), and the mobile phase was prepared through on-line mixing of methanol and an aqueous solution of 5 mM sodium acetate and 0.01 mM disodium EDTA (pH 4.5). The flow rate of the mobile phase was 1 mL/min. Methanol was linearly increased from 5% (*v*/*v*) to 10% (*v*/*v*) between 0 and 7 min of the separation and from 10% (*v*/*v*) to 30% (*v*/*v*) between 9 min and 20 min of the separation. The injection volume was 40 μL. The detection parameters were as follows: an excitation wavelength of 280 nm, an emission wavelength of 345 nm, sensitivity change from 5 to 8 at 10 min. of the separation, and a detector temp. of 45 °C. Analysis of chromatograms was performed using Chromeleon 6.8 software (Dionex, Sunnyvale, CA, USA). The limits of quantification (*S*/*N* ratio of 10:1 and RSD ≤ 15%) for 5-HTRP and 5-HT were 5 pg per injection, for TRP and 5-HIAA—10 pg per injection, and for 5-HTOL, NAS, 5-MTAM, 5-MIAA, 5-MTOL and MLT—2.5 pg per injection. The intraday precision (RSD of peak area) was below 3%, and the interday precision was below 4%.

### 4.4. Statistical Analysis

The data were analyzed by two-way ANOVA with the age of embryos and the sampling time as factors. Duncan’s test was used as a post hoc procedure. Because of extremely large changes in the content of 5-hydroxy- and 5-methoxyindoles during development, it was possible that some time-dependent differences in level of indoles in the youngest embryos were not detected by two-way ANOVA. Therefore, one-way ANOVA with the sampling time as factor and Duncan’s test as a post hoc procedure was additionally performed for the data from ED 14, ED 16, and ED 18 embryos. A value of *p* < 0.05 was considered as significant. The analyses were performed using Dell Statistica 13 (Version 13.1 PL, Dell Inc., Tulsa, OK, USA).

## 5. Conclusions

The embryonic ontogeny of 5-hydroxyindoles and 5-methoxyindoles biosynthesis pathways in the goose pineal organ is composed of three phases. During the first phase, the pineal organ produces and metabolizes 5-HT, but it lacks the ability to perform 5-HT acetylation and methylation of 5-hydroxyindoles. The process of 5-HT acetylation is turned on in the second phase, which begins around ED 16. During the third phase, starting from ED 18, the pineal organ produces 5-methoxyindoles, including MLT. The levels of 5-hydroxyindoles and 5-methoxyindoles increase with the age of the embryos and are highest between ED 26 and ED 28. The pineal organ of goose embryos is characterized by a high content of 5-HT, compared to a 16-day-old chicken; however, this level is much lower than that in the organs of a 14-week-old goose. This finding suggests that a species-specific type of indole metabolism is determined during embryonic life and further matures after hatching. Diurnal rhythmicity develops very early in the embryonic pineal organ of the goose when the eggs are incubated under a 12 h light:12 h dark schedule. The pineal contents of 5-HTRP, 5-HT and 5-HIAA show diurnal variations in ED 14 embryos, being higher during scotophase than during photophase. The diurnal variations in the pineal content of NAS, MLT and 5-MIAA were noted for the first time in ED 20 embryos. The courses and amplitudes of diurnal rhythms change during development. Obviously, two processes are responsible for generation of the diurnal rhythms of 5-hydroxyindoles and 5-methoxyindoles in the embryonic pineal organ: (i) hydroxylation of TRP and (ii) acetylation of 5-HT. The pattern of diurnal profiles of 5-methoxyindoles are also controlled at the methylation step.

The obtained results clearly show that the intensive development of MLT synthesis pathway occurs in the goose pineal organ during the last 4–6 days before hatching. This strategy ensures that the circadian timing system is ready to work properly at the time of hatching. Our data provide a strong argument that MLT rhythm is an important part of endocrine regulation in chicks of precocial water birds, starting from the first hours of post-hatching life.

## Figures and Tables

**Table 1:** Parameters of eggs incubation.

Days of Incubation	Temperature (°C)	Relative Humidity (%)	Ventilation (%)
1–16	37.8	55	40
17–26	37.4	60	60
27	37.0	60	60
28	37.0	65	60

## Abbreviations

TRP	tryptophan
5-HTRP	5-hydroxytryptophan
5-HT	serotonin
NAS	*N*-acetylserotonin
5-HIAA	5-hydroxyindole acetic acid
5-HTOL	5-hydroxytryptophol
5-MIAA	5-methoxyindole acetic acid
5-MTOL	5-methoxytryptophol
5-MTAM	5-methoxytryptamine
